# Seeing through noise in power laws

**DOI:** 10.1098/rsif.2023.0310

**Published:** 2023-08-30

**Authors:** Qianying Lin, Mitchell Newberry

**Affiliations:** ^1^ Theoretical Biology and Biophysics, Los Alamos National Laboratory, Los Alamos, NM, USA; ^2^ Michigan Institute for Data Science, University of Michigan, Ann Arbor, MI 48109-1382, USA; ^3^ Department of Biology, University of New Mexico, Albuquerque, NM, USA; ^4^ Department of Human Behavior, Ecology and Culture, Max Planck Institute for Evolutionary Anthropology, Leipzig, Saxony, Germany; ^5^ Center for the Study of Complex Systems, University of Michigan, Ann Arbor, MI, USA

**Keywords:** Pareto distribution, self-similarity, scale-free, tail index, fat tail, extreme value

## Abstract

Despite widespread claims of power laws across the natural and social sciences, evidence in data is often equivocal. Modern data and statistical methods reject even classic power laws such as Pareto’s law of wealth and the Gutenberg–Richter law for earthquake magnitudes. We show that the maximum-likelihood estimators and Kolmogorov–Smirnov (K-S) statistics in widespread use are unexpectedly sensitive to ubiquitous errors in data such as measurement noise, quantization noise, heaping and censorship of small values. This sensitivity causes spurious rejection of power laws and biases parameter estimates even in arbitrarily large samples, which explains inconsistencies between theory and data. We show that logarithmic binning by powers of *λ* > 1 attenuates these errors in a manner analogous to noise averaging in normal statistics and that *λ* thereby tunes a trade-off between accuracy and precision in estimation. Binning also removes potentially misleading within-scale information while preserving information about the shape of a distribution over powers of *λ*, and we show that some amount of binning can improve sensitivity and specificity of K-S tests without any cost, while more extreme binning tunes a trade-off between sensitivity and specificity. We therefore advocate logarithmic binning as a simple essential step in power-law inference.

## 1. Introduction

Power laws are ubiquitous in nature and arise from many distinct mechanisms [1–4]. Probability distributions with power-law tails are called heavy tailed and fat tailed, because they admit extreme events far more often than normal random variables. The probability to observe a large event scales inversely with the *α*th power of the magnitude *x* as

1.1 $$\text{Prob}(\mathrm{obs}.>x)={\left(\frac{x}{x_{m}}\right)}^{-\alpha },\;x>x_{m}>0,$$

and so forecasting extreme events depends sensitively on estimates of the exponent *α*. The self-similar nature of power laws, also called scale-free or scale-invariant, further enticingly suggests mechanisms that operate with no preferred scale across many orders of magnitude, such as criticality in earthquakes [5] or preferential attachment in networks [6,7]. Hence it is often of interest whether a given phenomenon follows a power law. Power laws have been observed or claimed in systems as diverse as turbulence, physiology [8], terrorism [9], baby name popularity [10] and cities [11–13], while the standards for scientific and statistical justification have varied widely over time and between fields.

Broad potential application and varying standards have led researchers to underscore an ongoing need for disseminating better statistical methods [14–16]. Ad hoc methods give different answers, which has complicated ongoing discussion of the relevance of power laws in observational studies [12,16–18]. Because power laws appear as straight lines on log–log plots, fitting by linear regression has a long history [19]. Regression methods continue to be applied and improved [20] but offer only qualitative assessment of goodness of fit and suffer from known pitfalls [21]. Maximum-likelihood and Kolmogorov–Smirnov (K-S) statistics come with a well-developed theory with clear guarantees [22,23] and have been advocated for power laws in recent decades [14,15]. Nonetheless, problems still arise in practice. Maximum-likelihood estimators (MLEs) give erroneous estimates in the presence of partial censorship [24], measurement error [25] and quantization noise [26], while K-S tests for goodness of fit often reject power laws [27], including such classic power laws as the Pareto law for wealth [28] and the Gutenberg–Richter law for earthquakes [19], given high-quality data [15]. Rejection by the K-S test indicates discrepancy between the model and the data, but the discrepancies are often routine data quality problems [12,25,27] rather than relevant features of the phenomena under investigation such as transient dynamics [29], finite size effects [30,31] or deviations from criticality [32].

We explain that failures of MLEs and K-S tests often stem from innocuous errors that the statistics do not account for, including random measurement noise, data coarsening, quantization, combining data of varying accuracy and scale-dependent censorship. These errors often go unnoticed because in normal statistics, many errors average out without affecting the shape of the distribution or biasing estimates of the mean. In power-law distributions, however, even small normal measurement errors qualitatively change the shape of the distribution and bias estimates of the exponent.

Logarithmic binning, by powers of *λ* > 1, mitigates these errors by averaging data within each scale. Whereas normal statistics reduce the effects of noise by averaging, we show that power-law statistics can reduce the effects of noise by aggregating data at each power of *λ*. Logarithmic binning creates equally spaced bins on the log scale, and so evenly covers the range of power-law data. While the usual MLEs and K-S statistics are perturbed by arbitrarily small errors, statistics based on the binned data are only affected by errors large enough to move data between bins. Greater *λ* increases the proportional threshold errors must exceed to affect the binned data, and so increases robustness to small errors. Logarithmic binning is furthermore the only scheme to preserve self-similarity in the underlying data [26,33]. In particular, if the input data follow a power law, the binned data follow a discrete power law distribution with the same exponent, leading to closed-form solutions for the likelihood and MLE [26]. Logarithmic binning therefore improves estimates and conclusions from noisy data by preserving information about the shape of the distribution over orders of magnitude while ignoring small errors within each scale.

## 2. Results

**Figure 1.**
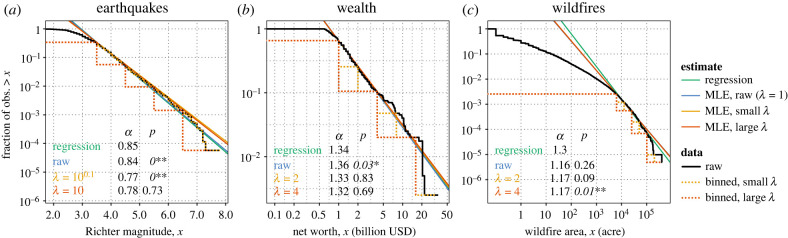
Binning reveals that measurement errors and quantization noise bias maximum-likelihood estimates of the slope *α* and alter conclusions of goodness-of-fit tests in (*a*) earthquake magnitudes and (*b*) wealth. For (*c*), wildfire data that does not follow a power law, coarse binning (*λ* = 4) correctly rejects the power law when raw data falsely accepts it. Tables list regression estimates, MLEs and goodness-of-fit test *p*-values with and without binning with ratio *λ*. *Italic* *p*-values reject the power law with *p < 0.05 or **p < 0.01.

We first show three empirical case studies (figure 1) where trivial errors in data qualitatively affect conclusions about power laws and binning increases reliability. Under the pure power law null hypothesis, results with and without binning should be mutually consistent [34] and the choice of *λ* is mostly arbitrary. In our cases, the results depend significantly on which binning scheme is used, which indicates deviations from the pure power law. The cases differ, however, in whether the deviations indicate trivial errors versus potentially real features of the underlying process. In wealth and earthquakes, binning removes deviations due to quantization and measurement artefacts, after which the data are consistent with a scale-free process. In wildfire area, binning makes tests more likely to reject the power law, suggesting real deviations in the underlying process.

### 2.1. Methods overview

Here, we provide a minimal description of the methods and notation, reserving a detailed description for the electronic supplementary material, S1, while we discuss analysis and validation in §3. We estimate *α* using MLEs and test goodness of fit using the K-S statistic, in accordance with widely accepted best practices [15]. We bin the input data by truncation, replacing each data point *x*_*i*_ with the binned value $\lfloor x_{i}\rfloor_{\lambda }=x_{m}\lambda^{\left\lfloor \log_{\lambda }x_{i}/x_{m}\right\rfloor }$, so that the data takes on the discrete set of values *x*_*m*_*λ*^*k*^ for *k* = 0, 1, 2, …. The likelihood for power-law data binned this way is also a power law [26],

2.1 $$L_{\lambda }(x|\alpha )=\prod_{i}(1-\lambda^{-\alpha }){\left(\frac{\lfloor x_{i}\rfloor_{\lambda }}{x_{m}}\right)}^{-\alpha }.$$

Binned data nonetheless entail different MLEs and null distributions of the K-S statistic [34]. We therefore notate the MLEs ${\hat{\alpha }}_{\lambda }$ to emphasize that the MLE depends on the binning ratio *λ* and we use an appropriate bootstrapped null distribution of K-S statistics [14,35] (electronic supplementary material, S1). The limit *λ* → 1^+^ corresponds to no binning, and so we use the symbol ${\hat{\alpha }}_{1}$ for the usual continuous MLE [15] and *λ* = 1 to denote the raw data. Naively applying ${\hat{\alpha }}_{1}$ to binned data results in substantially biased estimates even for very fine binning with *λ* close to 1 [26,34].

### 2.2. Earthquakes

Earthquake magnitudes are historically believed to follow a power law, where the exponent is the Gutenberg–Richter *b*-value [5,19,31]. The earthquake dataset [15] contains 17 450 samples ranging over seven orders of magnitude from 0.5 to 7.8 on the Richter scale. Traditional Richter magnitudes inherently constitute a case of logarithmic binning, because they record the base-10 logarithm of wave amplitudes to two digits of precision [36]. Exponentiating these magnitudes onto their natural scale results in a dataset with discrete values proportional to integer powers of *λ* = 10^0.1^ ≈ 1.26 ranging over seven orders of magnitude. Partial censorship is known to complicate inference below the magnitude of completeness *m*_*c*_ [37], which depends on the density of the earthquake sensor network. We choose *x*_*m*_ = 10^3.5^, or magnitude 3.5, to visibly exceed *m*_*c*_, retaining 5910 data points.

The results in figure 1*a* and table 1 substantially depend on binning. We conduct inference with no binning (*λ* = 1), *λ* = 10^0.1^ and *λ* = 10. Choosing *λ* = 10^0.1^ matches the quantization of the Richter scale. This ‘binning’ preserves the input data exactly but entails a different MLE and null distribution of K-S statistics. The raw and fine-binned empirical distributions therefore overlap in figure 1*a* but imply different interpretations of the data. The coarse binning *λ* = 10 represents increments of 1.0 in magnitude and divides the data into five bins.

**Table 1.** Power-law hypothesis test results compared with expectations

	expectation	raw data	fine bins	coarse bins
earthquakes	*power law* [19]	reject	reject	*accept*
wealth	*power law* [28]	reject	*accept*	*accept*
wildfires	*not power law*	accept	marginal	*reject*

*Italic* results are consistent with expectations.

Differences in the results point to features of the data rather than the underlying mechanisms. The effect of quantization noise on MLEs is evident from the significant difference between the continuous MLE ${\hat{\alpha }}_{1}=0.837\pm 0.006$, which falsely assumes no quantization, and the MLE that accounts for the quantization ${\hat{\alpha }}_{10^{0.1}}=0.77\pm 0.01$. Quantization noise therefore biases ${\hat{\alpha }}_{1}$ by approximately 0.07 or 9%, comparable to the 12% bias observed in other earthquake data [26]. Because errors in *α* occur in the exponent, however, this 9% correction predicts a nearly twofold higher rate of earthquakes larger than 7.8, the largest that appear in the dataset.

Such quantization noise would be sufficient reason for K-S tests to reject the pure power law, yet the test also rejects the power law when accounting for quantization with *λ* = 10^0.1^ (*p* < 0.01). This is because special magnitudes 3.0, 3.5, 4.0, 4.5, …, contain an excess of observations, as can be observed in the histogram (electronic supplementary material, figure S1) and quantile–quantile (QQ) plot (electronic supplementary material, figure S2). This excess creates a statistically significant association between ‘round’ numbers and higher-than-adjacent frequencies (*p* < 0.0001, *χ*^2^ test, 1 d.f.). The dataset therefore appears to combine low-precision data quantized at magnitude increments of 0.5 or 1.0 with high-precision data at increments of 0.1. The K-S test then understandably rejects the power law when the preponderance of data at increments of 0.5 cannot be explained by chance. Coarse binning with *λ* = 10 ignores these excesses and the test then accepts the power law with *p* = 0.73.

Taking *λ* = 10 still offers ample power to reject alternative distributions, as we reject the power law given fewer bins and far less data in other examples. Therefore, by taking its flaws into account, we conclude that the dataset provides strong evidence that the earthquake magnitudes follow a power law, in agreement with long-held conventional wisdom in geology (table 1).

### 2.3. Wealth

Vilfredo Pareto—for whom the continuous power law distribution is named—observed power laws in wealth and income as early as 1895 [28]. Measuring wealth remains a difficult and important problem in economics, social science and government [38]. The Forbes 400 list (subsequently The Forbes World’s Billionaires) famously tracks the wealth of the world’s richest people. Prior work, however, found the 2003 dataset inconsistent with a power law [15]. We analyse the dataset’s 261 billionaires, using *x*_*m*_ = 10^9^ and binning by powers of *λ* = 2 and *λ* = 4 (table 1 and figure 1*b*).

The Forbes lists are subject to limitations on accurate measurements of extreme wealth as well as data collection artefacts. The quantization scheme is inconsistent, with values truncated to the nearest 5 million or 100 million depending on whether net worth exceeded one billion. Among the billionaires, all three MLEs and regression give mutually consistent *α* estimates, which suggests overall conformity to a power law, but K-S tests disagree depending on binning.

Bumps evident in the tail distribution (figure 1*b*) and QQ plot (electronic supplementary material, figure S2) between 4 billion and 10 billion cause the K-S test on the raw data to reject the power law with *p* = 0.03, suggesting further inconsistency in representation of values. Indeed, many billionaires are listed has having either 4 or 5 billion while others are distinguished between 4.4, 4.7 or 4.9. In a case of heaping or attraction to particular values, higher-precision values occur noticeably more often just shy of round numbers, for example 6.9, 8.9, 9.7 and 9.8. Any larger amount of binning such as *λ* = 2 or 4, however, effectively removes these artefacts (figure 1*b*, dotted lines) and causes the test to accept the power law, vindicating Pareto’s seminal observation.

### 2.4. Wildfires

A wildfire dataset demonstrates a contrasting example in which binning leads hypothesis tests to reject the power laws when they otherwise would not, despite removing information from the sample. Some models of wildfire spread exhibit power-law scaling, for example through self-organized criticality [39]. However, the power law distribution is not borne out in this empirical data.

The data include 203 784 precise observations of wildfire area over 10 years in the USA recorded consistently to ±0.1 acre with up to six digits of precision over a range from 0.1 to 412 050 acres. A basic visual inspection of the distribution in figure 1*c* reveals smooth and substantial curvature clearly inconsistent with a power law, even allowing for error and statistical variation. Methods for fitting *x*_*m*_ have nonetheless been devised to locate a fraction of the tail distribution that is consistent [15]. This method finds power-law behaviour only in the highest 0.3% of the data—520 data points.

This *x*_*m*_ = 6324 acres indicates either a cusp beyond which the distribution follows a power law, or else the limit at which there is insufficient statistical power to reject the power law due to too little data. We conduct the inference with this *x*_*m*_ for *λ* = 1, 2 and 4. The MLEs ${\hat{\alpha }}_{1}$, ${\hat{\alpha }}_{2}$ and ${\hat{\alpha }}_{4}$ give mutually consistent results but differ from the regression estimate, which hints at persistent curvature beyond *x*_*m*_.

The tests, however, are more likely to reject the power law the coarser the binning. The test accepts the power law in the raw data (p=0.26) as stipulated by the calibration of *x*_*m*_. Binning with increasing *λ*, however, drives the test to reject the power law, with fine bins giving *p* = 0.09 and coarse bins ultimately rejecting the power law with *p* = 0.01. The coarse binning with *λ* = 4 corresponds to four bins, the last with only one data point. K-S statistics for binned data are therefore capable of rejecting the power law with 10-fold less data and fewer bins than in the earthquake data with *λ*=10.

While the earthquake and wealth examples show how raw K-S statistics ascribe undue weight to smoothness in the data, conversely, if smoothness is removed from consideration, the test must ascribe more weight to the shape of the distribution over powers of *λ*. Binning therefore causes K-S statistics to reveal curvature in the empirical distribution by allowing across-scale curvature to stand out from within-scale noise.

## 3. Analysis

The foregoing cases illustrate how binning can be used in conjunction with careful data analysis to explain trivial deviations from a power law and support more robust inference, or else to reject the power law based on consistently contra-indicative evidence. For the remainder, we show how binning provides a tool to control for error and improve inferences, by exploring the effects of errors and binning in simulated cases where the errors and alternative distributions are known.

Ideal prescriptions for binning would depend on the nature of errors, possible alternative distributions, the size of the data, and the scientific question. We assume the errors and alternatives are unknown in practice; otherwise, better methods exist such as modelling the error distribution [40]. Nonetheless, binning makes relatively weak assumptions about the error model, and therefore supports general strategies to control for unspecified error and mitigate bias in inferences.

### 3.1. Effects of normal and lognormal noise

We investigate particular cases of normal and lognormal noise where samples from the power-law equation (1.1) with *x*_*m*_ = 1 are convolved with unbiased additive normal noise with variance $\sigma_{+}^{2}$ or multiplicative lognormal noise with variance $\sigma_{\times }^{2}$, then truncated again at *x*_*m*_ for inference. This scheme represents several common sources of error: additive and proportional measurement error, censorship of small values, error in estimating *x*_*m*_ (the tail fraction [24,41]), and contamination from a bulk distribution [42]. The convolution creates measurement error, while the truncation creates both a case of censorship—because data reduced below *x*_*m*_ by noise are removed from the sample—and a bulk distribution distinct from its tail—because applying normal or lognormal error to power law samples yields a unimodal distribution peaked near *x*_*m*_. Normal error is furthermore a pessimistic case for binning. The quantization and heaping observed in the empirical cases can be completely removed by coarser binning, whereas normal error is always capable of large adjustments with some probability, and so no *λ* can completely prevent normal error from moving data between bins. In this way, our noisy power laws are chosen as representative and difficult cases of dirty data.

**Figure 2.**
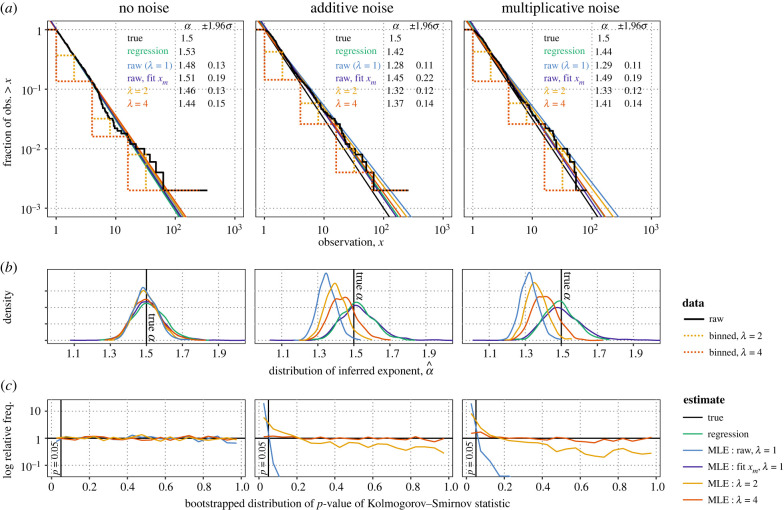
Simulated error in power-law data biases estimates of the exponent *α* and causes goodness-of-fit tests to reject the power-law distribution. (*a*) Samples from perfect data (left) and data with additive and multiplicative noise truncated at *x*_*m*_ = 1 (middle: normal (0, 0.2), right: lognormal (0, 0.2)). Solid black curves show the sample empirical tail distributions; dotted lines show the same after logarithmic binning with *λ* = 2 and *λ* = 4. Although the noise is only barely visible, it visibly biases estimates of the slope −*α* relative to the true *α* = 1.5 ((*a*) straight line slopes, (*b*) smoothed histograms). Logarithmic binning with *λ* = 2 or *λ* = 4 brings the slope estimates ${\hat{\alpha }}_{\lambda }$ closer to the true value. (*b*) Distributions of the slope estimates over 1000 samples of size *n* = 500 illustrate a trade-off between accuracy and precision, where the most precise estimation methods are also the least accurate. The conventional method of allowing the fit to exclude data by increasing *x*_*m*_ (purple) produces accurate estimates at the cost of the highest variability. (*c*) Distributions of Kolmogorov–Smirnov *p*-values are uniform when the data comes from the hypothesized distribution. However, noise biases *p*-values (middle, right) so that *p* <0.05 more than 98% of the time. Binning attenuates noise, brings *p*-value distributions closer to uniformity and eventually restores stipulated false positive rates.

Figure 2 shows the effect of normal and lognormal noise with *σ* = 0.2 on MLEs and K-S statistics in samples of size 500 with *α* = 1.5. The noise is barely visually discernible on log–log plots of the tail distribution, but nonetheless biases the raw MLE ${\hat{\alpha }}_{1}$ by 10–13% on average depending on noise treatment. This bias substantially exceeds the approximately 3.5% standard deviation of the estimator, causing the 95% confidence intervals (CIs) to exclude the true value 68% of the time, and corresponds to a twofold higher prediction for the frequency of extreme events larger than those observed in the dataset.

The theory of maximum likelihood guarantees that ${\hat{\alpha }}_{1}$ is the most efficient [22] and indeed the distribution of ${\hat{\alpha }}_{1}$ is the most sharply peaked (figure 2*b*). However, ${\hat{\alpha }}_{1}$ is correspondingly the most sensitive to errors while all other estimates are more robust to noise. Coarser binning, with larger values of *λ*, brings the MLEs ${\hat{\alpha }}_{\lambda }$ closer to the true value on average. For additive noise, binning with *λ* = 2 approximately halves the bias relative to the standard deviation of the estimator (*z*-score of the true value) to 1.56. Using *λ* = 4 further reduces the *z*-score of the true value to 0.84 and CIs exclude the true value only 15% of the time. In this example, regression minimizes the total root mean squared (RMS) error due to bias and variability, ${\left(\Sigma_{i}{\left(\alpha -{\hat{\alpha }}_{i}\right)}^{2}\right)}^{1/2}$, although regression is known to produce biased estimates in other cases [20,21] such as earthquakes above.

The raw K-S *p*-values are sensitive detectors of noise (figure 2*c*). For pure samples, the distribution of *p*-values is uniform. Uniform *p*-values control the false positives rate because, for a given stipulated allowable false positive rate *ρ*, uniformity implies that *p* < *ρ* exactly *ρ* fraction of the time. With noise, however, the *p*-values reject the power law at *ρ* = 5% nearly all of the time (additive: 98%, multiplicative: 99%). Binning with sufficiently large *λ* restores a uniform distribution of *p*-values (figure 2*c*), making the hypothesis test robust to noise. With *λ* = 2, rejection rates fall to 23% and 34%, while with *λ* = 4 the rejection rates are statistically consistent with ρ=5% over 1000 trials (additive noise: 5.4%, multiplicative noise: 6.1%).

The validity of the *p*-values with and without binning depend on the scientific question. If the question is whether the data follow the continuous power law exactly, then the raw *p*-values with *λ* = 1 are valid and the test is adept at rejecting power laws with even small amounts of noise. If the question is whether the data follow a power law allowing for some experimental noise, then the raw *p*-values fail to control the false positives rate and nearly always falsely reject the power law even when the data do follow a power law. The *p*-values for binned data are valid for either question by controlling the false positives rate when *λ* is sufficently large. The distribution of binned data (equations (1.1), electronic supplementary material, equation (S3)) is therefore a better null distribution for the more practical latter question.

A classical strategy for removing the effects of censorship of small values, contamination from a bulk distribution, and error in estimating the tail fraction or *x*_*m*_ is to simply increase *x*_*m*_, as more extreme values are more likely to follow the limiting tail distribution and larger values are less likely to be contaminated by error [15,24,41,42]. This strategy also applies here, and so we compare its effectiveness against binning with *x*_*m*_ fixed at 1. Fitting *x*_*m*_ by minimizing K-S distance, the power law [15,41] selects *x*_*m*_ = 1.4 ± 0.8 (±1.96 s.d.) and ${\hat{\alpha }}_{1}=1.52\pm 0.21$ for additive error and *x*_*m*_ = 1.5 ± 0.9, ${\hat{\alpha }}_{1}=1.50\pm 0.23$ for multiplicative error (figure 2*b*). This strategy minimizes bias by throwing away the most contaminated data, typically 20–25%, but comes at the cost of nearly doubling the variability of the estimator. Binning with *λ* = 4 and *x*_*m*_ = 1 performs slightly better in representing the true value—with RMS error of 0.09 and 0.11 for additive and multiplicative noise in contrast to 0.11 and 0.12 when fitting *x*_*m*_. Fitting *x*_*m*_, on the other hand, minimizes bias. Neither method alone outperforms linear regression by either standard in this example.

### 3.2. Power-accuracy trade-off in maximum-likelihood estimators

**Figure 3.**
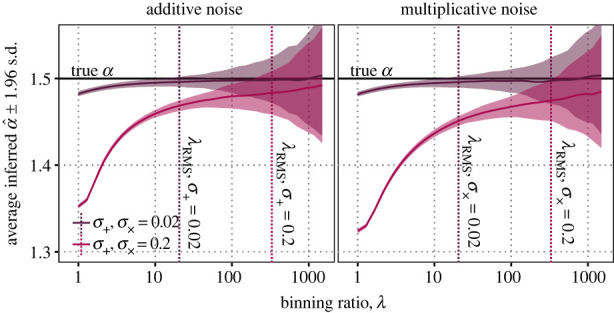
The binning ratio *λ* tunes a trade-off between accuracy and precision of the estimator ${\hat{\alpha }}_{\lambda }$ in data with additive and multiplicative noise. Here, we simulate large datasets (*n* = 1 000 000, median range *r* = 12 500, 200 replicates for each *λ*) with additive and multiplicative noise with variance *σ*_+_ and $\sigma_{\times }=0.02\;\mathrm{and}\;0.2$. Increasing *λ* brings the average estimates ${\hat{\alpha }}_{\lambda }$ closer to the true *α* = 1.5 but also increases the variance of the estimator. The binning ratio *λ*_RMS_ which minimizes the total mean squared error on the estimate due to bias and variability (vertical dotted lines) depends on the type and magnitude of error.

Much as increasing *x*_*m*_ removes potentially erroneous data at the cost of statistical power, binning also removes information from the sample, inducing a trade-off between accuracy and power in estimation. Increasing *λ* increases variability on the estimator (electronic supplementary material, S1),

3.1 $$\sigma_{{\hat{\alpha }}_{\lambda }}^{2}=\frac{{\left(\lambda^{{\hat{\alpha }}_{\lambda }}-1\right)}^{2}}{\left(n\lambda^{{\hat{\alpha }}_{\lambda }}\log^{2}\lambda \right)},$$

but potentially reduces unknown bias, depending on errors in the data. The binning ratio *λ* can therefore trade unknown bias for a calculable increase in variability. Better trade-offs are achievable with larger sample size, as larger samples both reduce variability on the estimator and expand the range of the data, allowing larger *λ*. Increasing *λ* with sample size, by holding variability constant, therefore allows larger samples to decrease bias due to error, in contrast to the typical cases of estimation in which bias is independent of sample size. We investigate the trade-off numerically in samples of size *n* = 1 000 000 (200 replicates; *α* = 1.5; *σ*_+_, $\sigma_{\times }=0.02,0.2$). Figure 3 shows a clear trade-off between accuracy and precision as a function of *λ* in our normal and lognormal noisy power-law data.

We then compute the *λ*_RMS_ that minimizes the RMS error in representing the true value for each noise treatment. This *λ*_RMS_ ranged from 20 to 330, corresponding to dividing the data into two to four bins. In comparison with the proportional range of the data *r* := max_*i*_ *x*_*i*_/*x*_*m*_, the fraction of log-range covered by the first bin log *λ*_RMS_/log *r* varied only from 0.32 to 0.61 (median *r*: 12 500, $\sqrt{r}\;:\;111$). That is, the *λ*_RMS_ that best represents the true value typically divided the data into two or three bins despite a 10-fold difference in error magnitudes.

### 3.3. Sensitivity and specificity of Kolmogorov–Smirnov tests

**Figure 4.**
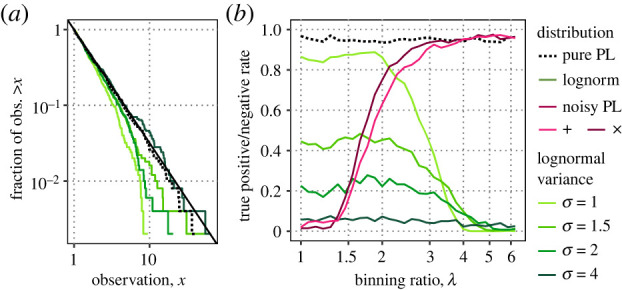
For (*a*) lognormal tail samples chosen to resemble the power-law, (*b*) binning entails no decrease in statistical power below a threshold of *λ*. (*a*) Samples (n=500, 400 replicates) from the tail of the lognormal distribution can approximate power-law samples increasingly well by increasing the lognormal variance $\sigma_{\ln}^{2}$ from 1 to 4. (*b*) The test sensitivity on lognormal samples is not substantially affected by binning provided *λ* < 2, corresponding to dividing the data into at least four bins, whereas specificity on noisy power-law data increases dramatically with higher *λ* < 2. For 2 < *λ* < 4, there is a trade-off between sensitivity and specificity, while for *λ* > 4, corresponding to only two or three bins, the test is useless, as it accepts the power law regardless of the underlying distribution.

Hypothesis testing generally involves a trade-off between sensitivity and specificity. The test should reject the null hypothesis when it is false (sensitivity) and accept it when it is true (specificity). Statistical variability makes perfect performance on both measures impossible, whereas sensitivity often can be increased at the cost of specificity and vice versa. While greater *λ* generally increases specificity by ignoring errors in power-law data, it is possible for greater *λ* to increase sensitivity as well, as observed in the wildfires example. Here, we show in simulations of power law, noisy power law and lognormal distributions that *λ* may increase specificity with no cost to sensitivity, or else tune a trade-off between sensitivity and specificity (figure 4).

We evaluate the performance of hypothesis testing using lognormal samples truncated below *x*_*m*_ = 1 as an alternative, non-power-law distribution. The lognormal distribution is heavy-tailed and so its extremes can be difficult to distinguish from a power law [15]. Both tail distributions can appear straight on a log–log plot, but the lognormal has curvature that depends on its parameters (figure 4*a*). The truncated lognormal probability density function is

$$f\;(x)=\left\{ \begin{array}{cc} \frac{l(x)}{Z}\; & x\geq x_{m}\;\\ 0\; & \mathrm{otherwise}\; \end{array}\right.,\;l(x)=\frac{\exp[(-{\left(\ln x-\mu \right)}^{2})/2\sigma_{\ln}^{2}]}{x\;\sigma_{\ln}\sqrt{2\pi }},$$

where *l*(*x*) is the usual lognormal density function and *Z* is the normalization $\int_{x_{m}}^{\infty }l(u)\;du$. Its three parameters are the mean *μ*, variance $\sigma_{\ln}^{2}$ and tail threshold *x*_*m*_. As *σ*_ln_ goes to infinity, the curvature diminishes and *f*(*x*) converges to a power law with a fixed *α* = 1. The lognormal tail can therefore approximate a power law with any *α* > 1 arbitrarily well for sufficiently large but finite *σ*_ln_. We set *x*_*m*_ to 1, use *σ*_ln_ to adjust curvature, and finally choose *μ* so that the slope is equal to *α* = 1.5 at *x*_*m*_ to approximate the small power law samples of figure 2 where *n* = 500.

We find that binning entails no loss of sensitivity or statistical power, provided *λ* divides the data into at least four bins (figure 4*b*). The sensitivity itself depends strongly on the curvature of the lognormal. At *σ*_ln_ = 1, the most curved, sensitivity is approximately 90% whereas at *σ*_ln_ = 2, 28% is the best attainable. However, sensitivity was independent of *λ* for *λ* < 2 for any given curvature. The median range of all samples was 12.8, and so *λ* = 2 typically puts the maximum data point in the fourth bin, [8,16) (figure 4*a*).

Increasing *λ* removes noise, and so increases the specificity of the test, when we take rejections of the power law due to noise as false positives. The specificity given pure continuous power-law data is always 1 − *ρ* = 0.95, whereas specificities on noisy data are not always 0.95, but instead depend on *λ* due to noise biasing K-S *p*-values. The range 2 < *λ* < 4 shows a trade-off between sensitivity and specificity (figure 4*b*), in which increasing *λ* decreases sensitivity to reject lognormal tails and increases specificity for accepting comparable noisy power-law samples with $\sigma_{+},\sigma_{\times }=0.2$ (cf. electronic supplementary material, section (a)).

Overall, increasing *λ* increases specificity at no cost to sensitivity up to a point. This much is always desirable. Further increases in *λ* sacrifice sensitivity for specificity, which may be desirable, up to such *λ* as the test becomes useless because it is unable to reject the power law more often than *ρ* under any circumstances. In general, these thresholds of *λ* depend on sample size, type and magnitude of errors in the data, and possible alternative distributions, but reasonable guesses can be made based on number of bins.

## 4. Discussion

We argue here that logarithmic binning can reveal noise in otherwise power-law-distributed data, attenuate bias in estimation and reduce false positives in hypothesis tests. Binning thereby provides a tool for the empiricist to see through noise in power-law distributed data.

We have shown in empirical and simulated cases that errors such as quantization, data coarsening, combining data of different precision, measurement error, censorship of small values, and error in estimating *x*_*m*_ can all affect inferences. This implies that MLEs and K-S statistics that assume a pure, continuous power law cannot be trusted when the data may contain small errors. However, logarithmic binning, using the discrete power law of equation (1.1), attenuates some of these errors or removes them from consideration.

When different binning schemes reveal inconsistencies among estimators, or contrasting conclusions of hypothesis tests, at most one of the contradictory inferences can be accurate, and more careful data analysis may support or discredit one or another inference. Binning the data with different *λ* thereby reveals important features of the data. If hypothesis tests accept the power law at *λ* = 1, estimates at other *λ* are mutually consistent and independent of *λ*, and hypothesis tests accept the power law approximately 1 − *ρ* fraction of the time regardless of *λ* [34], then there is no evidence of deviation from a pure power law. In that case, *λ* = 1, that is, no binning, offers the most precise estimates. However, if binning reveals inconsistency among the estimators ${\hat{\alpha }}_{\lambda }$ or causes hypothesis tests to reject the power law, as in the case of wildfires, this is evidence against the power law that is only revealed through binning.

If hypothesis tests reject the power law at *λ* = 1, however, it may be due simply to errors in the data rather than any underlying phenomena. Inference at different *λ* may reveal threshold beyond which the test accepts the power law, as in earthquakes, wealth, and the simulated errors of figure 4. Thresholds provide clues to features of the data, such as the quantization and special values of earthquake data, the heaping in the wealth distribution, or the type and magnitude of error in simulation. We explore how the threshold depends on *σ*_+_, $\sigma_{\times }$, *α* and sample size for our cases of normal and lognormal error and truncation in electronic supplementary material, section (a) and figure S5 over a wide range of physically reasonable *α* from 0.5 to 3.5.

Windows of consistency among estimators and tests over a range of *λ* are assurance against cherry-picking results in pseudoreplicated multiple hypothesis testing such as *p*-hacking [43]. Conversely, results that hold only for a particular *λ* and fail to replicate at different *λ* should be considered suspect. This caution applies equally to results for *λ* = 1, and so replicating results for other *λ* > 1 should be incorporated into common practice. Finally, estimation is also predicated on the assumption that the data come from a power law, and so estimates using a *λ* for which the hypothesis test fails are suspect.

### 4.1. Choosing values of *λ*

Considerations involved in choosing values of *λ* include the nature of the errors in the data, possible alternative distributions, the sample size and the scientific question. For example, binning should always respect the quantization in the data, if present. If the data are logarithmically quantized at some *λ*_0_ as in the earthquake data, only integer powers of the quantization, $\lambda =\lambda_{0}^{k}$, should be considered. If the data are quantized to a linear or inconsistent scale, *λ* should be chosen large enough to make the differences negligible, or more complicated binning schemes should be devised (cf. [34]).

Estimating *α* requires *λ* to be within the proportional range of the data *r*, so that the MLE ${\hat{\alpha }}_{\lambda }$ is well defined (electronic supplementary material, equation (S4)). At the two extremes of the range 1 < *λ* ≤ *r*, ${\hat{\alpha }}_{1}$ maximizes bias while minimizing statistical variability, whereas ${\hat{\alpha }}_{r}$ minimizes bias but maximizes variability. Neither extreme is desirable unless the data is perfect, in which case there is no bias and ${\hat{\alpha }}_{1}$ minimizes variability. On imperfect data, equally dividing the logarithmic range of the data using $\lambda =\sqrt{r}$ balances bias against variability to some degree, and is reasonable unless more detailed information is available. Indeed, in the numerical experiments of figure 3, increasing noise by a factor of 10 only doubles log *λ*_RMS_/log *r*, so that $\lambda =\sqrt{r}$ achieves comparable performance to *λ*_RMS_ over a wide range of error magnitudes. This *λ* corresponds to a minimum of bins, however, which offers the hypothesis test no information, and so these estimates are predicated on other belief that the data do indeed follow a power law. Other reasonable prescriptions for estimation are to minimize bias subject to an allowable statistical error using equation (2.1) or choosing *λ* to greatly exceed a known magnitude or proportional error.

Hypothesis testing imposes different limits on *λ* because more bins are required to distinguish the shapes of alternative distributions. Increasing *λ* reduces the chance of a false rejection due to trivial errors, but also might reduce capacity to reject alternative distributions. Numerical experiments indicate that choosing *λ* < *r*^1/4^ entails no loss of power to reject lognormal data, whereas choosing *λ* > *r*^0.4^ offers no power to reject alternatives. Binning the data into at least five bins by choosing *λ* up to *r*^1/4^ is therefore a prescription for detecting curvature over the range of the data, whereas detecting deviations over a limited range such as roll-off [31,44] or censorship of small values [37] would require more bins within the interval of interest. Coarser binning than *r*^1/4^ may sacrifice sensitivity for a possible increase in specificity, but binning beyond *λ* = *r*^0.4^ is likely to have no sensitivity and is therefore useless.

## 5. Conclusion

MLE and K-S tests offer strong mathematical guarantees, but only if the error model is well specified. The fragility of these methods in power laws has thus far been underappreciated. Loose specification of errors is convenient in practice because the details of error sources are often unknown. Regression offers a one-size-fits-all model which is robust to error (cf. Figure 2, [20]), but not without other drawbacks [15,21]. Detailed specification of errors provides a more accurate solution [40] but requires careful work that is not always feasible. Using the discrete distribution of equation (1.1) makes strictly weaker assumptions about the data than the usual continuous power law and is therefore more tolerant of error while retaining the clarity and benefits of likelihood methods and K-S tests.

Considerable discussion in recent decades has questioned the validity and value of power laws [15–17]. Often, however, these studies have left zero tolerance for error [26,27]. Binning offers a way to re-evaluate the quality of empirical power laws with some allowance for trivial experimental or data collection error. The same debate has sometimes conflated establishing the existence of a power law with measuring its exponent, whereas these are distinct scientific questions that require different assumptions and contextual information. Binning likewise offers different models within the same family suited to the different goals and assumptions of parameter estimation and hypothesis testing.

Binning offers a complement and alternative to classical methods of removing error by increasing *x*_*m*_ [15,24,41,42]. Both binning and increasing *x*_*m*_ are appropriate to different circumstances and are not mutually exclusive. Increasing *x*_*m*_ cannot remove the effects of quantization and heaping that occur in our empirical cases of wealth and earthquakes. Indeed, fitting *x*_*m*_ leads to rejecting the power law in both cases and a substantially biased ${\hat{\alpha }}_{1}$ = 0.64 for earthquakes [15]. Furthermore, there are often scientific reasons for specifying *x*_*m*_, such as direct knowledge of magnitude of completeness *m*_*c*_ in earthquakes [37] or the imaging resolution in vascular data [8]. In such cases, fitting *x*_*m*_ can lead to ignoring valid data and a sacrifice of statistical power.

We conclude that logarithmic binning—combined with appropriate MLEs and goodness-of-fit tests—offers a rough but effective control for common data errors that otherwise make power-law inference unreliable. Common errors necessitate the development and wider application of more robust inference methods in the many scientific contexts in which power laws arise. Given the ubiquity of power laws in nature and errors in data, we urge the incorporation of binning into standard practice for power-law inference.

## Data Availability

Data obtained from Aaron Clauset [15] from the GitHub repository: https://aaronclauset.github.io/powerlaws/data.htm. The data are provided in electronic supplementary material [45].
